# Localized Induction Heating for Crack Healing of AISI 1020 Steel

**DOI:** 10.3390/ma19030451

**Published:** 2026-01-23

**Authors:** Aprilia Aprilia, Zixuan Ling, Vincent Gill, Nicholas Chia, Martyn A. Jones, Paul E. Williams, Wei Zhou

**Affiliations:** 1 Rolls-Royce@NTU Corporate Laboratory, Nanyang Technological University, 65 Nanyang Drive, Singapore 637460, Singapore; 2 School of Mechanical and Aerospace Engineering, Nanyang Technological University, 50 Nanyang Avenue, Singapore 639798, Singapore; 3 Rolls-Royce Singapore Pte Ltd., 1 Seletar Aerospace Crescent, Singapore 797565, Singapore; 4 Rolls-Royce PLC, P.O. Box 31, Derby DE24 8BJ, UK

**Keywords:** induction heating, crack healing, eddy current, current crowding, current detouring

## Abstract

This study investigates crack healing of AISI 1020 steel using localized induction heating with a pancake coil. A wire-cut slit sample and a repetitive-bent sample containing fine cracks were subjected to induction heating. Geometrical changes in the slit and cracks before and after heating were evaluated. Healing of fine cracks and local melting of the slit tip were observed. Numerical simulations were conducted to understand the current flow, current density distribution and Joule heating behavior within the samples. Results showed that current detours around cracks and concentrates at crack tips during induction heating. This enables the ability of induction heating to selectively locate and treat cracks effectively. Localized induction heating using a pancake coil enhances the crack-healing effectiveness by providing a non-singular current flow direction within the material. It also offers the flexibility to treat a specific localized region in a component. While localized induction heating demonstrates strong potential for crack-healing applications, its effectiveness is primarily limited to fine surface cracks.

## 1. Introduction

The application of electric current in manufacturing and remanufacturing of metallic materials, commonly referred to as electric-assisted manufacturing (EAM), has been extensively studied over the past several decades. A substantial body of literature has demonstrated that the introduction of direct or pulsed electric current can significantly alter deformation behavior, microstructural evolution, and thermomechanical response in metals. In deformation-based processes, EAM is widely reported to reduce flow stress, enhance ductility, and improve formability, thereby enabling lower processing loads and expanding forming limits [1,2].

Early pioneering investigations by Okazaki, Conrad, Stepanov, and co-workers established the fundamental scientific basis of EAM by systematically examining the interaction between electric current and metallic materials [3,4,5,6,7]. Their studies revealed that electric current can induce a range of beneficial effects, including stress relaxation, accelerated recrystallization, enhanced diffusion, and current-assisted phase transformations. Subsequent research has expanded these findings across a wide range of materials and processing routes, confirming that EAM can be effectively applied to forming, joining, surface modification, and microstructural control of metals.

More recently, increasing attention has been directed toward the use of electric current not only to facilitate deformation, but also to repair and heal defects such as cracks, pores, and damage accumulated during service. This emerging research direction exploits the localized thermal, electromigration, and current-assisted plasticity effects generated by electric current to promote defect closure and microstructural recovery. Beyond its microstructural effects, EAM offers several important process-level advantages for repair and remanufacturing applications. One key advantage is accelerated repair, as the rapid and localized energy input associated with electric current enables defect healing to be achieved within significantly shorter processing times compared to conventional furnace-based heat treatments. In addition, EAM allows highly localized treatment, which minimizes thermal exposure of the surrounding bulk material and reduces the risk of global microstructural degradation or distortion.

Another important benefit of EAM-based repair is its energy efficiency and process flexibility, as electrical energy is delivered directly to the targeted region rather than heating the entire component. This localized and selective heating capability also enables in situ or on-site repair, reducing the need for component disassembly and lowering downtime in industrial applications. These process advantages make electric current-assisted repair particularly attractive for extending the service life of metallic components subjected to localized damage.

Representative studies by Zhou et al. [8,9,10] demonstrated that high-density pulsed current could effectively mitigate quenching cracks in steel components and repair cracked saw blades, while simultaneously improving mechanical performance. Similarly, Song et al. [11,12] reported that electric current treatment could heal microcracks generated by severe plastic deformation through localized recrystallization and microstructural rearrangement. Collectively, these works illustrate that electric-current-assisted repair is a viable and rapidly developing approach for extending the service life of damaged metallic components.

The crack-healing concept has also been explored using induction-based approaches, where eddy currents are generated within the material without direct electrical contact. Compared to direct electric current treatment using physical electrodes, induction heating offers a non-contact and more flexible processing route, eliminating issues related to electrode positioning and contact resistance. Studies employing induction coils for nondestructive testing have demonstrated that induced eddy currents are highly sensitive to internal and surface flaws, producing measurable variations in response to defect location and geometry [13,14]. Yang et al. [15,16] applied eddy current-based treatment to heal fatigue cracks in 1045 steel tubes, where a significantly elevated temperature at the crack tip facilitated crack closure and re-bonding. Within the same research group, Xu et al. [17,18] further investigated similar crack-healing phenomena in non-ferrous metal tubes using pulsed eddy current treatment with solenoid coils.

Despite these advances, existing induction-based crack-healing studies have largely focused on solenoid coils and axisymmetric geometries, where the entire component circumference is exposed to eddy-current heating. Localized crack-healing using pancake-type induction coils, which enable targeted heat delivery to specific damaged regions, has not yet been systematically investigated. Such a configuration is particularly attractive for repairing large or geometrically complex components, where full-field induction heating may be impractical. Building on prior work demonstrating the effectiveness of localized high-frequency induction heating for rapid brazing and coating densification [19,20,21], the present study aims to explore the capabilities of localized high-frequency induction heating for crack-healing applications. Two types of specimens, a wire-cut slit sample and a repetitive-bent sample containing fine cracks, will be subjected to localized induction heating using a pancake coil. Geometrical changes in the slit and crack regions before and after treatment will be examined to assess the extent of defect mitigation. In addition, numerical simulations will also be performed to analyze current flow behavior, current density distribution, and Joule heating characteristics within the specimens. The potential and limitations of localized induction heating for crack-healing applications will be discussed.

## 2. Materials and Methods

### 2.1. Wire-Cut Slit of AISI 1020 Steel

**Figure 1:**
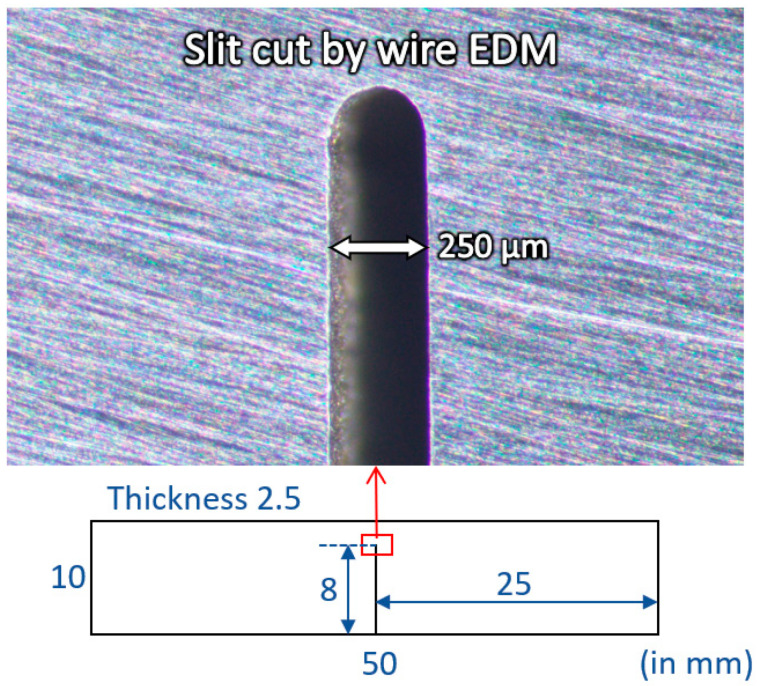
Thin wire-cut slit sample of AISI 1020 steel representing a thin crack.

A thin wire-cut slit with a width of 250 µm was created by a wire electrical discharge machining (EDM V500G, Ichi Seiki, Singapore) on a rectangular AISI 1020 steel sample, as shown in Figure 1. The dimensions of the sample coupon were 50 mm × 10 mm × 2.5 mm, and its chemical composition is listed in Table 1. This thin wire-cut slit was intended to simulate a thin crack in steel. A slit length of 8 mm was cut perpendicularly from the midpoint of one of the long edges. Before induction heating, the wire-cut sample was ground using SiC papers up to 2000 grits.

### 2.2. Repetitive Bending Crack of AISI 1020 Steel

**Figure 2:**
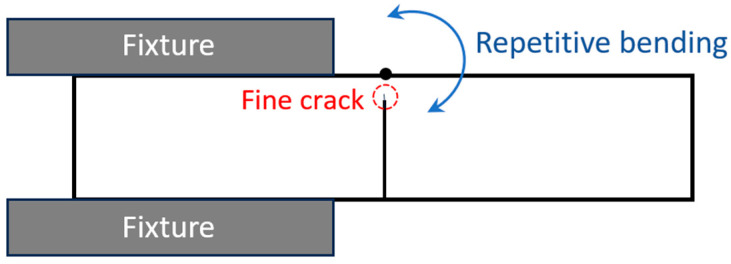
Fine cracks in 1020 steel produced by repetitive bending.

Finer cracks with widths of less than 50 µm were also produced for investigation. These cracks were generated through a manual bending process. One end of the wire-cut steel sample was rigidly clamped in a bench vice, while the free end was manually bent back and forth using a hammer. The bending was performed for five cycles to introduce plastic deformation and initiate fine surface cracks near the bent region. Figure 2 illustrates the bending direction applied to the sample. Following the bending process, scanning electron microscopy (SEM) micrographs of the resulting cracks were obtained and used as reference images prior to the induction heating treatment.

### 2.3. Induction Heating

**Figure 3:**
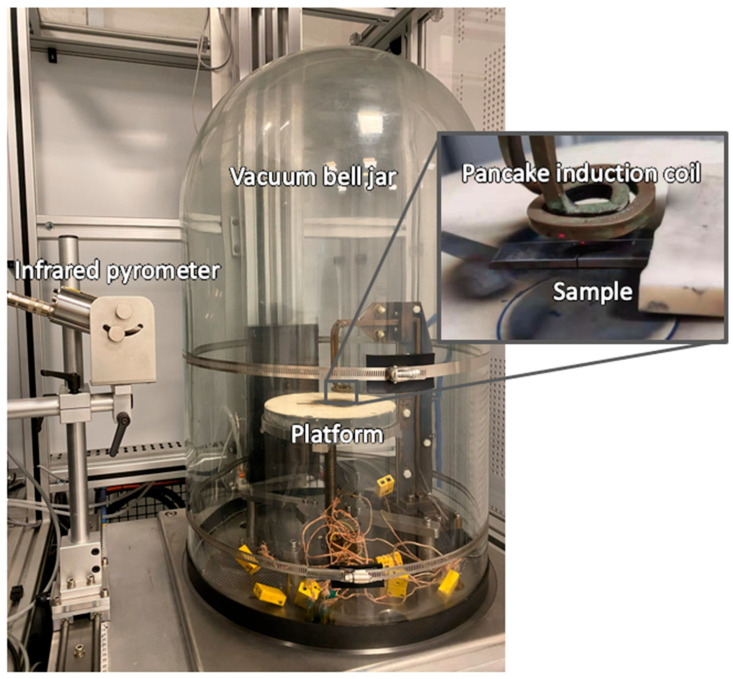
Localized induction heating setup with a pancake induction coil.

Localized induction heating was performed using a high-frequency induction heater equipped with a water-cooled spiral pancake coil, as shown in Figure 3. The operating frequency was set to 107 kHz. The pancake coil was made of copper and had an outer diameter of 30 mm. A vacuum bell jar was used to minimize surface oxidation, with vacuum pressure maintained at 5 × 10^−4^ torr. The induction heating system operated in a closed-loop mode, in which an infrared pyrometer was used to monitor and regulate the sample temperature. The infrared pyrometer was directed at the top surface of the sample near the tip of the wire-cut slit. The measured temperature was fed back to the control system to adjust the induction power, thereby maintaining a constant target temperature for the specified heating duration. In this study, each sample was positioned beneath the coil and heated to 900 °C for 5 min. After heating, the sample was allowed to cool inside the vacuum chamber for 5 min, after which argon gas was introduced to further cool the sample to room temperature.

### 2.4. Sample Characterization

Crack observations before and after induction heating were conducted using a stereo microscope (SZX7, Olympus, Tokyo, Japan) and a scanning electron microscope (JOEL 5600LV, Tokyo, Japan). For microstructure analysis, the samples were ground using SiC papers up to 4000 grits, followed by polishing with 1 µm diamond suspension and 0.25 µm colloidal silica suspension. Etching was performed using a 3% Nital reagent (3 mL HNO_3_ and 97 mL ethanol) for 10 s.

### 2.5. Numerical Simulations

To analyze the current distribution within the samples during induction heating, numerical simulations were conducted using Ansys Maxwell 3D 2020 R2 (Ansys, Canonsburg, PA, USA) with the Eddy Current Solver. The sample model was created using the same dimensions and material properties as the experimental specimen. The pancake coil was simplified to a ring-shaped model with an outer diameter of 30 mm, an inner diameter of 20 mm, and a thickness of 5 mm. The coil material was set to copper, consistent with the actual heating coil. The excitation frequency and current were set to 107 kHz and 97 A, respectively, matching the experimental conditions.

## 3. Results

### 3.1. Wire-Cut Slit Changes After Induction Heating

**Figure 4:**
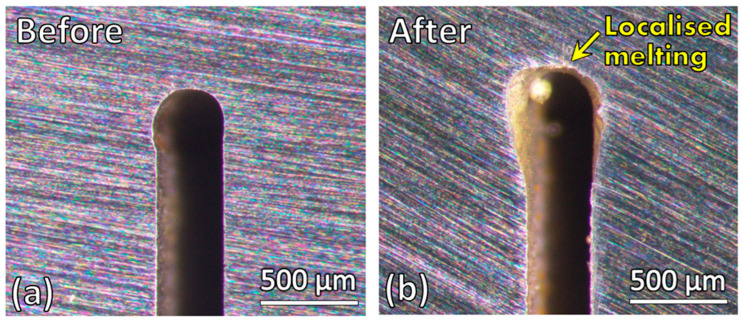
Stereo micrographs of the slit: (**a**) before induction heating, (**b**) after induction heating.

Figure 4 shows the stereo micrographs of the wire-cut slit before and after induction heating. A noticeable geometrical change at the slit tip is observed after heating. The tip became significantly enlarged, and a sphere of solidified metal formed near the tip region. These observations indicate that localized melting occurred at the slit tip during the induction heating process.

**Figure 5:**
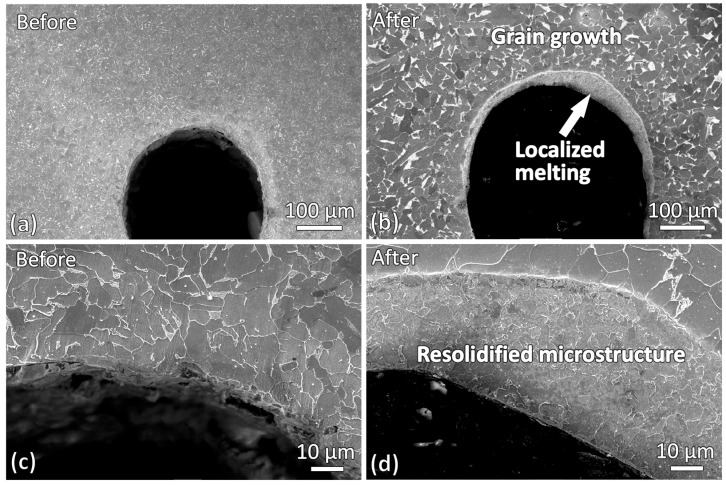
SEM micrographs showing microstructures near the slit tips at two different magnifications of the samples: (**a**,**c**) before induction heating and (**b**,**d**) after induction heating.

Microstructures of the wire-cut slit samples before and after induction heating were also obtained and compared. Figure 5 shows the microstructure near the slit tip region. In the bulk material, both the as-cut and induction-heated samples exhibit a ferrite-pearlite dual-phase steel microstructure. However, the induction-heated sample shows noticeably larger grain size, indicating grain growth in the bulk region during heating. Another prominent change is the presence of a layer of resolidified microstructure around the curved slit tip in the heated sample. This observation is consistent with the stereo micrographs in Figure 4, which showed evidence of localized melting at the slit tip during induction heating.

**Figure 6:**
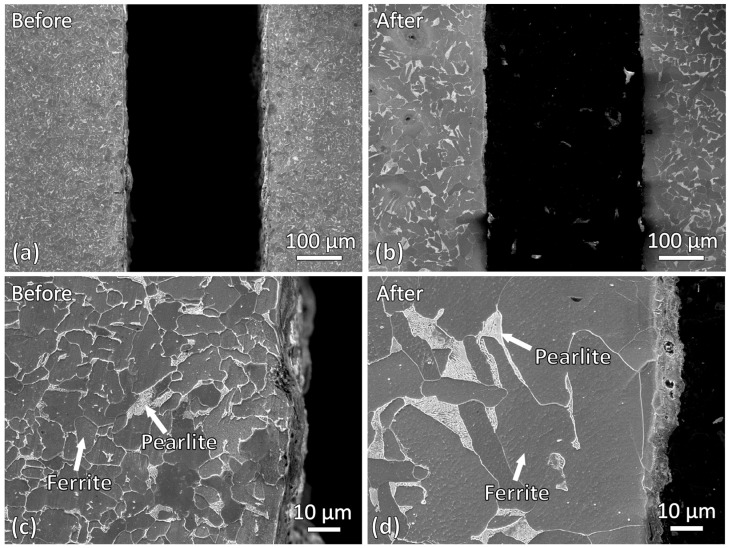
SEM micrographs showing microstructures near the slit tip of two different magnifications of the sample: (**a**,**c**) before induction heating and (**b**,**d**) after induction heating.

Microstructures of the regions adjacent to the slit edges before and after induction heating were also examined and compared as shown in Figure 6. Similar to the observations at the slit tip, significant grain growth was observed in the vicinity of the slit edges after induction heating. However, unlike the slit tip region, no resolidified layer was found near the slit edges. This indicates that, at the slit edges, only grain growth occurred, whereas localized melting did not take place.

**Figure 7:**
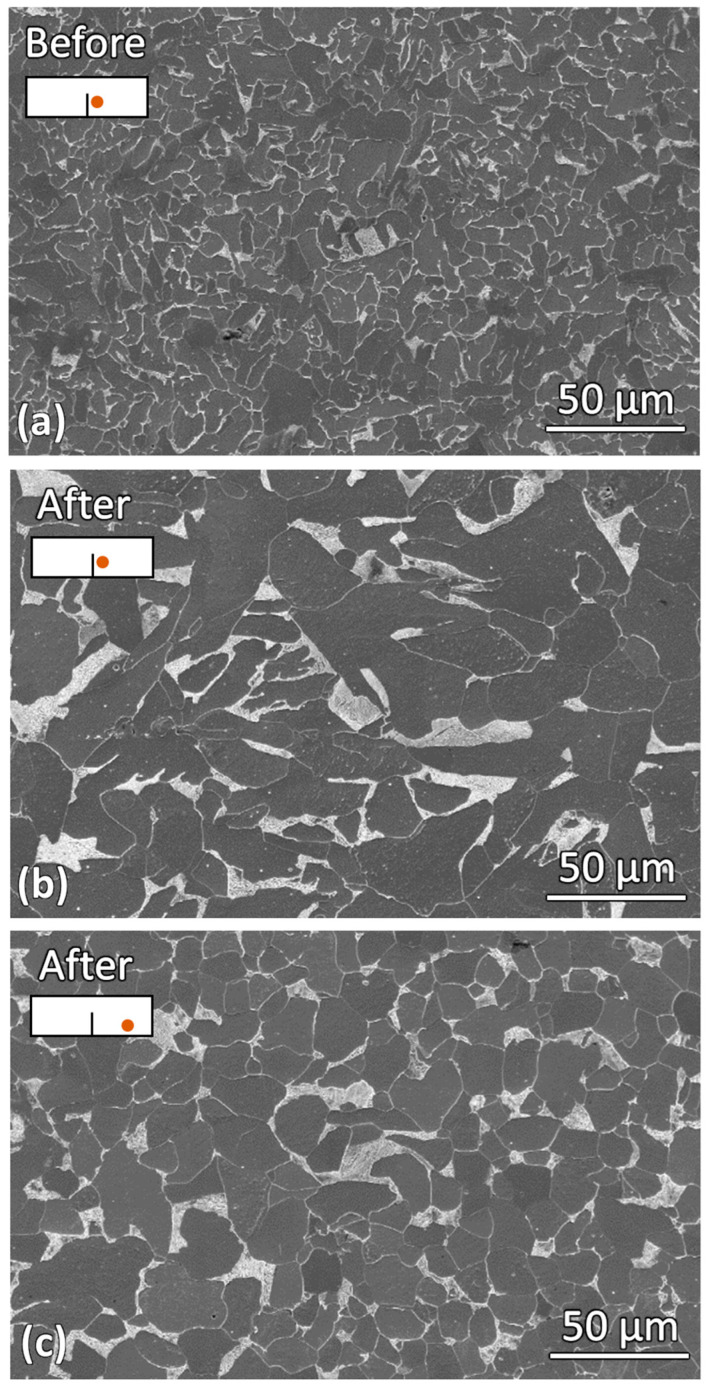
SEM micrographs showing the microstructures: (**a**) near the slit before induction heating, (**b**) near the slit after induction heating and (**c**) away from the slit after induction heating.

Grain growth was observed at both the slit tip and slit edges after induction heating. To determine whether this behavior was consistent throughout the entire sample, the microstructure in a region further away from the slit was also examined. Figure 7 compares the microstructures before induction heating, after induction heating near the slit, and after induction heating further away from the slit. A clear difference in grain size can be observed. The grain size of the sample before induction heating was the smallest. After induction heating, significant grain growth occurred, with grains near the slit region being larger than those further away. This indicates that the material near the slit may have experienced a higher temperature during induction heating. Furthermore, the localized melting observed at the slit tip, as shown in the SEM micrographs in Figure 5b,d, suggests that the slit tip experienced the highest temperature within the sample during heating.

Grain growth during induction heating is primarily governed by thermally activated diffusion processes and is strongly influenced by the peak heating temperature and soaking duration [22]. Higher temperatures and longer exposure times promote grain boundary mobility, resulting in accelerated grain coarsening, particularly in regions subjected to concentrated Joule heating. Other induction parameters, such as induction current and frequency, mainly influence the power input, heating rate, penetration depth and current-assisted dislocation mobility. However, their effects on grain growth are indirect and less significant than those of heating temperature and heating duration [22]. To mitigate excessive grain coarsening during induction-based crack healing, several strategies may be considered, including reducing the target peak heating temperature, shortening the holding time at elevated temperatures, and employing rapid heating-cooling cycles to minimize the duration at peak temperature. Ideally, the induction heating parameters should be optimized such that sufficient energy is locally concentrated in the crack vicinity to promote crack closure, while avoiding prolonged high-temperature exposure in other regions of the sample.

### 3.2. Fine Cracks from Repetitive Bending Changes After Induction Heating

**Figure 8:**
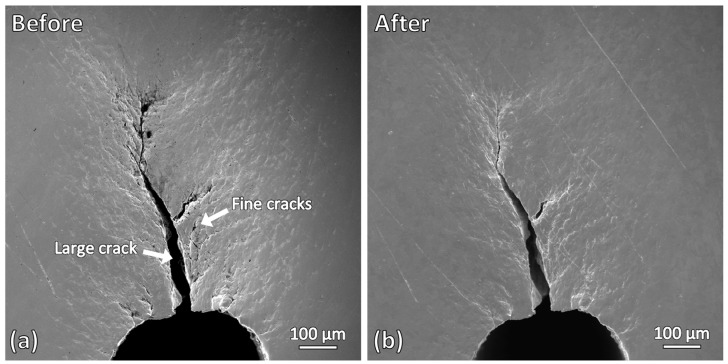
SEM micrographs of the fine cracks produced by repetitive bending: (**a**) before induction heating and (**b**) after induction heating.

Figure 8 shows the SEM micrographs of the fine cracks produced by repetitive bending, before and after induction heating. Noticeable changes can be observed. In the as-bended sample, one dominant crack with an opening width of approximately 30 µm is present, branching into numerous finer cracks with widths of less than 10 µm. After induction heating, the large crack remains unchanged; however, significant changes are observed in the fine cracks. Most of the fine cracks become barely visible after induction heating, indicating that crack healing has occurred in this sample. For the resolidified layer that was previously observed at the slit tip of the wire-cut slit sample (Figure 5b,d), it is not observed in this sample. This indicates that localized melting did not occur at the slit tip of this sample. The presence of cracks at the slit tip may have influenced this.

**Figure 9:**
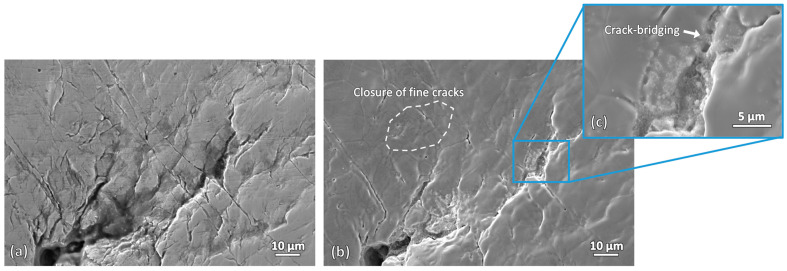
Higher-magnification micrographs of the fine cracks: (**a**) before and (**b**,**c**) after induction heating.

Figure 9 shows higher-magnification micrographs of the fine cracks before and after induction heating. Crack closure is clearly observed for fine cracks, particularly those with smaller crack widths. Most of the affected cracks have widths of less than 1 µm. However, not all fine cracks were successfully healed, with some cracks only partially bridged and not fully closed. Crack-bridging is observed in some cracks, indicating that the heating duration of 30 min used in this study may not be sufficient to achieve complete healing for these cracks. Crack-closure effectiveness can be further enhanced by increasing the induction parameters such as induction current, frequency, heating temperature, and heating duration [23].

## 4. Discussion

Experiment results showed that crack healing by induction heating did not occur in the wire-cut slit sample but did occur in the repetitive-bent sample with fine cracks. Localized melting was observed at the slit tip of the wire-cut slit sample, but not in the repetitive-bent sample, where extended cracks were present at the slit tip. To better understand the cause of these results, the current distribution within the samples during induction heating was evaluated using numerical simulation.

### 4.1. Detouring and Crowding of Eddy Current

**Figure 10:**
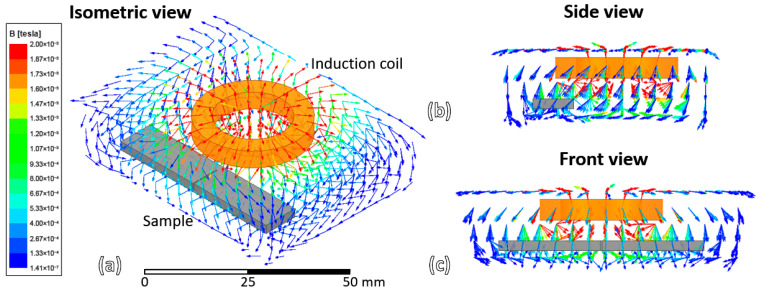
Magnetic field around the sample and induction coil (represented as a vector): (**a**) isometric view, (**b**) side view and (**c**) front view.

Numerical simulation of the induction heating process on the wire-cut slit sample was performed. Figure 10 shows the magnetic field around the sample and coil during the induction heating process at 0° phase.

**Figure 11:**
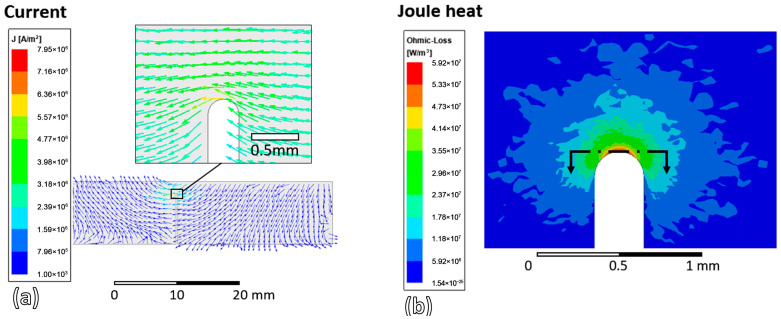
(**a**) Current and (**b**) Joule heat or ohmic-loss distribution on the upper surface of the sample near the slit tip region.

The induced eddy current density and its direction on the upper surface of the sample are shown in Figure 11a. It can be observed that the current density at the slit tip is several orders of magnitude higher than in the bulk material. This occurs because the current detours around the slit. As the two sides of the slit are separated, the current cannot flow directly across it; instead, it must flow around the slit tip, leading to “current crowding” in this region. Such current crowding commonly arises whenever there is a geometrical discontinuity within a sample. Figure 11b shows the joule heating (ohmic-loss) distribution near the slit tip. Joule heating refers to the conversion of the electrical energy carried by the induced current into heat due to the material’s resistivity, resulting in a local temperature rise [24]. Due to the current crowding at the slit tip, the joule heating or ohmic loss is correspondingly higher in the vicinity of the slit tip. These current crowding and joule heating contribute to the crack healing process.

### 4.2. Selective and Localized Healing of Cracks in Induction Heating

The current detouring and crowding phenomena that occur during induction heating are particularly beneficial for crack-healing applications. As the current flows through the material, it naturally concentrates in the vicinity of cracks due to the perturbation of the electric path, enabling cracks to be automatically located and treated during the process. The elevated current density near the crack region generates a localized thermal hot spot through Joule heating, which can reduce the local flow stress via thermal softening and thereby facilitate crack-face conformity and plastic accommodation during closure. Similar crack-related current detouring and crowding effects, accompanied by localized Joule heating, have been reported in studies of electrically assisted crack arrest and repair in metals [12,25]. In addition to Joule heating, the introduction of electric current has also been reported to reduce deformation resistance and increase apparent ductility or plasticity through electroplasticity-related mechanisms such as current-assisted dislocation motion, although the relative contribution of athermal effects and Joule heating remains a subject of ongoing debate [26]. Because the elevated current density and associated heating are highly localized around the crack, the treatment can preferentially promote plastic accommodation and crack closure near the defect while minimizing the thermal exposure of the bulk material.

**Figure 12:**
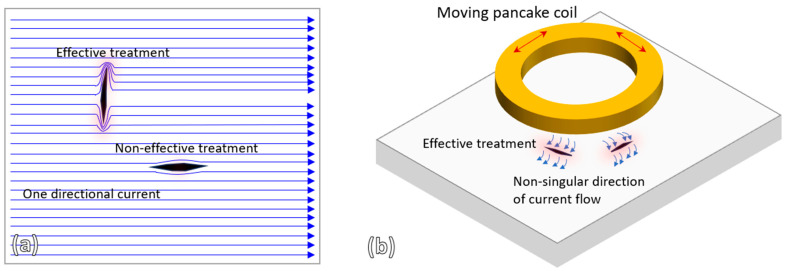
Illustrations of crack healing treatment effectiveness in: (**a**) one-directional current flow induction heating system, (**b**) non-singular direction of current flow in pancake coil induction heating system.

It should also be noted that the effectiveness of crack healing is influenced by the direction of current flow relative to the crack geometry. Figure 12a illustrates that a unidirectional current flow is effective only for cracks oriented in certain directions, which is typical in tubular induction heating systems. In contrast, the pancake induction coil used in this study generates a non-singular current flow pattern (see Figure 11a) and, when combined with coil movement during treatment, enhances the effectiveness of crack healing. Figure 12b illustrates how the current flow produced by a pancake induction coil can effectively treat cracks of various orientations.

**Figure 13:**
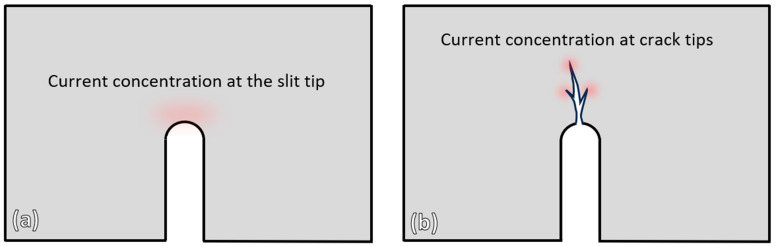
Illustrations of the high current concentration locations of: (**a**) wire-cut slit sample (**b**) repetitive bent sample investigated in this study.

The regions of high current concentration in both the wire-cut slit sample and the repetitive-bent sample investigated in this study are illustrated in Figure 13. In the wire-cut slit sample, the highest current concentration occurs at the slit tip, whereas in the repetitive-bent sample, it occurs at the tips of the fine cracks. This observation correlates with the experimental results, where localized melting was observed at the slit tip of the wire-cut slit sample, and crack healing of the fine cracks was observed in the repetitive-bent sample. In the case of the slit sample, the slit width was relatively large (approximately 250 µm), and the concentrated induced eddy current was insufficient to heal such a wide opening. However, for some fine cracks in the repetitive-bent sample, which had widths of less than 1 µm, successful crack healing occurred. These findings indicate that induction heating can be effectively used for crack healing, provided that the crack width falls within a suitable range and the induction parameters are appropriately selected.

Another limitation that should be noted is the skin-depth effect in induction heating. The skin-depth effect refers to the phenomenon in which the induced eddy current is confined to a certain depth from the material surface nearest to the coil. The skin depth δ (m) can be calculated using the following formula [21]:

(1) $$\delta =503\sqrt{\frac{\rho }{\mu_{r}F}}$$

where ρ is the electrical resistivity (Ωm) of the material, $\mu_{r}$ is the relative magnetic permeability of the material, and F is the excitation frequency (Hz) used for the induction heating. In this study, the ρ of AISI 1020 steel at room temperature is 1.59 × 10^−7^ Ωm [27]. The relative magnetic permeability of AISI 1020 steel ranges from 100 to 2000 [28], depending on the applied magnetic field strength. With an excitation frequency of 107 kHz, the skin depth of the induction heating process in this study is calculated to be approximately 0.014–0.062 mm at room temperature. As the sample temperature increases during heating, the skin depth also increases [20]. The skin depth can additionally be increased by reducing the excitation frequency. For materials with higher electrical resistivity and lower magnetic permeability, the skin depth will also increase.

Overall, the results of this study demonstrate that localized induction heating using a pancake coil is an effective approach for promoting crack closure in AISI 1020 steel, particularly for fine surface cracks. The observed crack closure and crack bridging are expected to improve mechanical performance by reducing stress concentration at crack tips, enhancing load transfer across partially closed cracks, and delaying crack propagation, thereby contributing to improved fatigue resistance and structural integrity. However, the effectiveness of this technique is subject to practical limitations. Crack healing is most effective for small, shallow surface cracks, as the induction heating process is governed by the skin-depth effect, which restricts current penetration and heat generation to near-surface regions. Consequently, localized induction heating is promising for mitigating early-stage surface damage. For deeper or wider cracks, further optimization of induction parameters and coil design should be explored to improve the current penetration and crack-closure effectiveness.

## 5. Conclusions

In this study, the use of localized induction heating with a pancake induction coil for crack healing in AISI 1020 steel was investigated. Localized induction heating was applied to a wire-cut slit sample and a repetitive bent sample. Micrographs of the samples before and after induction heating were obtained and analyzed. Numerical simulations were also conducted to examine the current flow, current density distribution, and Joule heating behavior within the samples during the process. The conclusions drawn from this study are as follows:

(1) Current crowding occurred at the slit tip of the wire-cut slit sample and at the fine crack tips of the repetitive-bent sample. This resulted in localized melting in the wire-cut slit sample and successful crack healing of fine cracks in the repetitive-bent sample.
(2) Current detouring and crowding of induction heating are useful for crack-healing applications, as cracks within the material can be automatically located and treated without significantly affecting the surrounding bulk material.
(3) Localized induction heating using a pancake coil has the advantage of providing a non-singular current flow direction within the material, enhancing the effectiveness of crack healing. The pancake coil configuration also offers the flexibility to only treat a specific localized region within a component.
(4) Crack healing by induction heating can effectively occur if the crack is located within the skin depth and if the crack width is not too large for the induction power used. Induction heating is highly suitable for crack-healing applications due to its ability to selectively locate and treat cracks; however, it is primarily effective for fine surface cracks.

## Figures and Tables

**Table 1:** Chemical composition (wt.%) of the AISI 1020 steel used.

C	Mn	Si	Ni	Cu	P	S	Fe
0.20	0.32	0.21	0.15	0.15	0.009	0.0042	Bal.

## Data Availability

The original contributions presented in this study are included in the article. Further inquiries can be directed to the corresponding author.
